# Immunohistochemical and Molecular Features of Melanomas Exhibiting Intratumor and Intertumor Histomorphologic Heterogeneity

**DOI:** 10.3390/cancers11111714

**Published:** 2019-11-02

**Authors:** Haider A. Mejbel, Sri Krishna C. Arudra, Dinesh Pradhan, Carlos A. Torres-Cabala, Priyadharsini Nagarajan, Michael T. Tetzlaff, Jonathan L. Curry, Doina Ivan, Dzifa Y. Duose, Raja Luthra, Victor G. Prieto, Leomar Y. Ballester, Phyu P. Aung

**Affiliations:** 1 Department of Pathology, The University of Texas MD Anderson Cancer Center, Houston, TX 77030, USA; mejbel@mail.etsu.edu (H.A.M.); drarudra@gmail.com (S.K.C.A.); drdineshpradhan@gmail.com (D.P.); ctcabala@mdanderson.org (C.A.T.-C.); pnagarajan@mdanderson.org (P.N.); mtetzlaff@mdanderson.org (M.T.T.); jlcurry@mdanderson.org (J.L.C.); dsivan@mdanderson.org (D.I.); vprieto@mdanderson.org (V.G.P.); 2 Department of Dermatology, The University of Texas MD Anderson Cancer Center, Houston, TX 77030, USA; 3 Department of Translational Molecular Pathology, The University of Texas MD Anderson Cancer Center, Houston, TX 77030, USA; dyduose@mdanderson.org (D.Y.D.); rluthra@mdanderson.org (R.L.); 4 Department of Hematopathology, The University of Texas MD Anderson Cancer Center, Houston, TX 77030, USA; 5 Department of Pathology and Laboratory Medicine, The University of Texas Health Science Center at Houston, Houston, TX 77030, USA

**Keywords:** melanoma, histophenotypic heterogeneity, tumor heterogeneity, next-generation sequencing, driver mutations

## Abstract

Melanoma is a heterogeneous neoplasm at the histomorphologic, immunophenotypic, and molecular levels. Melanoma with extreme histomorphologic heterogeneity can pose a diagnostic challenge in which the diagnosis may predominantly rely on its immunophenotypic profile. However, tumor survival and response to therapy are linked to tumor genetic heterogeneity rather than tumor morphology. Therefore, understating the molecular characteristics of such melanomas become indispensable. In this study, DNA was extracted from 11 morphologically distinct regions in eight formalin-fixed, paraffin-embedded melanomas. In each region, mutations in 50 cancer-related genes were tested using next-generation sequencing (NGS). A tumor was considered genetically heterogeneous if at least one non-overlapping mutation was identified either between the histologically distinct regions of the same tumor (intratumor heterogeneity) or among the histologically distinct regions of the paired primary and metastatic tumors within the same patient (intertumor heterogeneity). Our results revealed that genetic heterogeneity existed in all tumors as non-overlapping mutations were detected in every tested tumor (*n* = 5, 100%; intratumor: *n* = 2, 40%; intertumor: *n* = 3, 60%). Conversely, overlapping mutations were also detected in all the tested regions (*n* = 11, 100%). Melanomas exhibiting histomorphologic heterogeneity are often associated with genetic heterogeneity, which might contribute to tumor survival and poor response to therapy.

## 1. Introduction

Melanoma is one of the most heterogeneous neoplasms at both the histophenotypic and molecular levels. Melanomas often exhibit histophenotypic and molecular variations, both within the same tumor (intratumor) and between different tumors from the same patient (intertumor) [1]. The degree of histophenotypic variation can range from mild and focal changes to complete non-overlapping features of distinct histophenotypes, referred to as histophenotypic plasticity. This variation in histology and immunohistochemical expression of melanoma poses a diagnostic challenge that can alter disease stage and therapy.

At the molecular level, melanoma can be classified into four main types: v-Raf murine sarcoma viral oncogene homolog B (*BRAF*)*-*mutant, neuroblastoma RAS viral oncogene homology (*NRAS*)*-*mutant, neurofibromatosis type 1 (*NF1*)*-*mutant, and triple-negative or wild-type melanoma [1]. Mutations in *BRAF*, particularly *BRAF* p.V600E, are the most commonly detected mutations in melanoma [2,3,4,5,6], followed by mutations in *NRAS* [7,8,9,10] and *NF1* [11]. However, melanoma can exhibit intratumor and intertumor heterogeneity that may manifest at the genome, epigenome, or transcriptome level.

Although both histophenotypic plasticity and tumor heterogeneity of melanoma are well recognized, their relationship is not well explored. Therefore, we interrogated the molecular characteristics of a series of cutaneous melanomas exhibiting extreme intertumor and intratumor histomorphologic heterogeneity using region-specific next-generation sequencing (NGS) in an attempt to unmask the underlying tumor heterogeneity and to search for cancer-associated driver mutations that may play a helpful diagnostic or therapeutic role and may predict disease outcome.

## 2. Results

### 2.1. Clinicopathological Characteristics of Patients and Tumors Evaluated by NGS

The demographic, clinical characteristics, and disease outcome of the five patients are summarized in Table 1. Only one patient had a known history of therapy prior to sample collection (patient 4). Two patients (patients 1 and 2) had only a primary melanoma evaluated, and three patients (patients 3–5) had both a primary melanoma and a metastatic melanoma evaluated. All the primary melanomas were treated with wide local excision and sentinel lymphadenectomy. Complications included microsatellitosis (1.8 mm in largest dimension) identified in one patient (patient 3, 20%), regional nodal metastasis identified in three patients (patients 3–5, 60%), and distant metastases that developed during follow-up after initial surgical treatment in two patients (patients 1 and 4).

Histologically, all five primary melanomas exhibited a vertical growth phase that extended to the reticular dermis (Clark level IV; patients 2–4, 60%) and to the subcutaneous fat (Clark level V; patients 1 and 5, 40%), with a mean Breslow thickness of 5.14 mm (range: 1.2–12 mm) and mean mitotic rate of five mitoses per mm^2^ (range: 2–14 mitoses per mm^2^); however, no regression or brisk tumor-infiltrating lymphocytes was detected. Immunohistochemical studies revealed discordant patterns in expression of at least one melanocytic marker in four cases (patients 1–3 and 5, 80%), among whom a complete loss of at least one melanocytic marker was observed in two patients (patients 2 and 5, 50%). Alternatively, concordant pattern in expression of all the tested melanocytic markers was present in only one case (patient 4, 20%) (Table 2).

### 2.2. Histopathologic, Immunohistochemical, and Molecular Characteristics by Patient

**Figure 1:**
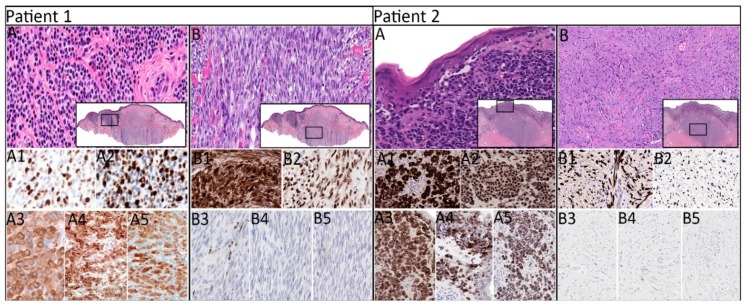
Histomorphologic and immunophenotypic of primary melanomas in patients 1 and 2. **Patient 1:** primary cutaneous melanoma with nevoid (**A**) and spindled (**B**) regions (H&E, ×400; insets, ×20) exhibiting diffuse expression of S100 (**A1**), SOX10 (**A2**), pan-melanocytic cocktail (**A3**), HMB-45 antigen (**A4**), and Melan-A (**A5**) in the nevoid region and diffuse expression of S100 (**B1**) and SOX10 (**B2**), scattered focal expression of pan-melanocytic cocktail (**B3**), and negative expression of HMB-45 antigen (**B4**) and MelanA (**B5**) in the spindled region (IHC, ×200). **Patient 2:** primary cutaneous melanoma with superficial epithelioid (**A**) and deep spindled (**B**) regions (H&E, ×400; insets, ×20) exhibiting diffuse expression of S100 (**A1**), SOX10 (**A2**), and pan-melanocytic cocktail (**A3**) and patchy expression of HMB-45 antigen (**A4**) and Melan-A (**A5**) in the epithelioid region and diffuse expression of S100 (**B1**) and SOX10 (**B2**) and negative expression of pan-melanocytic cocktail (**B3**), HMB-45 antigen (**B4**), and Melan-A (**B5**) in the spindled region (IHC, ×200). H&E, hematoxylin and eosin; IHC, immunohistochemistry.

In primary melanomas, the tumor of patient 1 had nevoid and spindled regions (Figure 1, patient 1, A and B, respectively). S100 and SOX10 exhibited diffuse expression in both regions, whereas pan-melanocytic cocktail exhibited diffuse expression in the nevoid region and focal expression in the spindled region. HMB-45 antigen and Melan-A were positive only in the nevoid region (Figure 1, patient 1, A1–A5 and B1–B5, respectively). *NRAS, TP53,* and *KDR* mutations were detected in both regions, whereas *APC* mutation was detected only in the nevoid region. The tumor of patient 2 had superficial epithelioid and deep spindled regions (Figure 1, patient 2, A and B, respectively). S100 and SOX10 were expressed diffusely in both regions, whereas pan-melanocytic cocktail, HMB-45 antigen, and Melan-A were expressed only in the epithelioid region (Figure 1, patient 2, A1–A5 and B1–B5, respectively). The same driver mutations of *TP53* and *CDKN2A* were detected in both regions, whereas *STK11* mutation was detected only in the epithelioid region.

**Figure 2:**
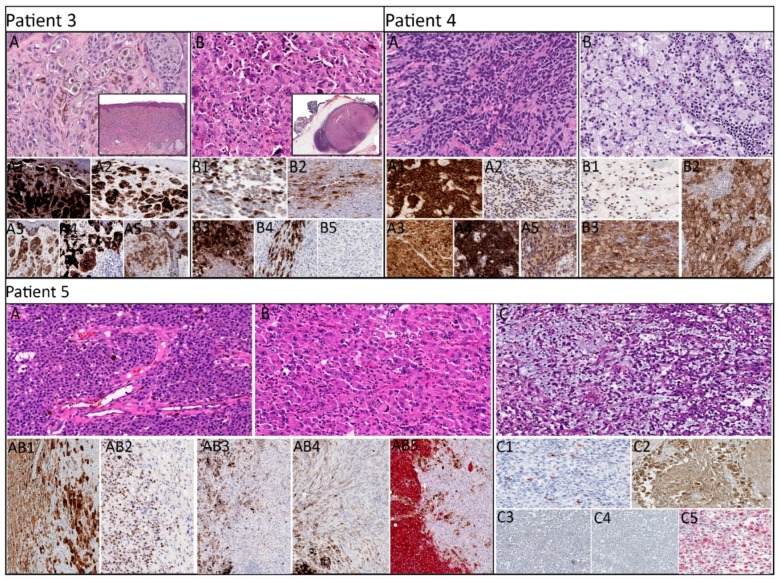
Morphologic and immunohistochemical expression of paired lesions in patient 3–5. **Patient 3:** primary cutaneous melanoma with epithelioid morphology (**A**) and histiocytoid morphology of intraparotid lymph node metastasis (**B**) (H&E, ×400; inset, ×20) exhibiting diffuse expression of S100 (**A1**), SOX10 (**A2**), pan-melanocytic cocktail (**A3**), HMB-45 antigen (**A4**), and Melan-A (**A5**) in the primary lesion and patchy expression of S100 (**B1**), SOX10 (**B2**), pan-melanocytic cocktail (**B3**), focal expression of HMB-45 antigen (**B4**), and negative expression of Melan-A (**B5**) in the metastatic lesion (IHC, ×200). **Patient 4:** primary cutaneous melanoma with epithelioid morphology (**A**) and right groin lymph node with metastatic melanoma with histiocytoid morphology (H&E, ×400) exhibiting diffuse expression of S100 (**A1**), SOX10 (**A2**), pan-melanocytic cocktail (**A3**), HMB-45 antigen (**A4**), and Melan-A (**A5**) in the primary lesion and diffuse expression of S100 (**B1**), SOX10 (not shown), pan-melanocytic cocktail (**B2**), and Melan-A (**B3**) in the metastatic lesion (IHC, ×200). **Patient 5:** primary cutaneous melanoma with epithelioid (**A**) and rhabdoid (**B**) morphologies and in-transit metastasis with myxoid morphology (**C**) (H&E, ×400) exhibiting diffuse expression of S100 (**AB1**), SOX10 (**AB2**), pan-melanocytic cocktail (**AB3**), HMB-45 antigen (**AB4**), and Melan-A (**AB5**) in the epithelioid region of primary lesion, patchy expression of S100 (**AB1**), negative expression of SOX10 (**AB2**), pan-melanocytic cocktail (**AB3**), and HMB-45 antigen (**AB4**), and focal expression of Melan-A (**AB5**) in the rhabdoid region of the primary lesion, and focal expression of S100 (**C1**), patchy expression of SOX10 (**C2**), negative expression of pan-melanocytic cocktail (**C3**) and HMB-45 antigen (**C4**), and weak expression of Melan-A (**C5**) in the metastatic lesion (IHC, ×200).

In paired (primary and metastatic) melanomas, the primary tumor of patient 3 exhibited epithelioid morphology (Figure 2, patient 3, A) and was diffusely positive for S100, SOX10, pan-melanocytic cocktail, HMB-45 antigen, and Melan-A (Figure 2, patient 3, A1–A5), whereas the metastatic lesion exhibited rhabdoid morphology (Figure 2, patient 3, B) with patchy expression of S100, SOX10, and pan-melanocytic cocktail, focal expression of HMB-45 antigen, and negative expression of Melan-A (Figure 2, patient 3, B1–B5). Molecular analysis revealed identical mutations of *BRAF, TP53,* and *ATM* in the primary and metastatic tumors, whereas *RAC1* mutation was identified only in the metastatic lesion.

In patient 4, the primary tumor exhibited epithelioid morphology, and the metastatic lesion exhibited foamy/balloon-like morphology (Figure 2, patient 4, A and B, respectively). S100, SOX10, pan-melanocytic cocktail, and Melan-A were diffusely expressed in both lesions, in addition to the positive expression of HMB-45 antigen in the primary lesion (Figure 2, patient 4, A1–A5 and B1–B3). NGS revealed mutation in *HNFA1* that was detected only in the primary lesion, and a mutation in *APC* was detected only in the metastatic lesion. In addition, the primary and metastatic lesions harbored identical *KIT* mutations.

In patient 5, the primary tumor exhibited a region with epithelioid morphology and a separate region with rhabdoid morphology, whereas the metastatic lesion exhibited myxoid morphology (Figure 2, patient 5, A–C, respectively). In the primary lesion, the epithelioid region exhibited diffuse expression of S100, SOX10, pan-melanocytic cocktail, HMB-45 antigen, and Melan-A (Figure 2, patient 5, AB1–AB5, left, respectively), and the rhabdoid region exhibited variable expression of S100 and Melan-A (Figure 2, patient 5, AB1 and AB5, right, respectively) with negative expression of SOX10, pan-melanocytic cocktail, and HMB-45 antigen (Figure 2, patient 5, AB2–AB4, right, respectively). Whereas the metastatic lesion was positive for S100 (focal), SOX10 (patchy), and Melan-A (weak) (Figure 2, patient 5, C1, C2, and C5, respectively) with a lack of pan-melanocytic cocktail and HMB-45 antigen (Figure 2, patient 5, C3 and C4, respectively). Identical mutations in *NRAS* and *TP53* were present in all three regions. A mutation in *CTNNB1* was present only in the epithelioid region of the primary tumor, and a mutation in *STK11* was present only in the metastasis.

### 2.3. Distribution of the Detected Driver Mutations

**Figure 3:**
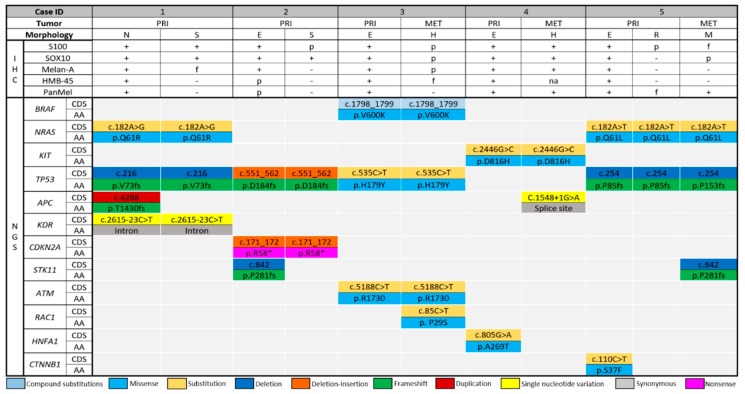
Next-generation sequencing of the histologically distinct regions revealed heterogeneous distribution of the detected cancer-associated driver mutations. In each mutated gene, specific changes detected at the nucleotide (CDS) and the peptide levels (AA), as well as sites of synonymous mutations, are illustrated. Abbreviations: PRI, primary; MET, metastatic; N, nevoid; S, spindled; E, epithelioid; H, histiocytoid; R, rhabdoid; M, myxoid; p, patchy; f, focal; na, not available.

The detected mutations are summarized in Figure 3. In the 11 histologically distinct regions of eight tumors retrieved from five patients, mutations were detected in 12 cancer-associated genes. Mutations in five of these genes (42%), namely, *APC*, *STK11*, *RAC1*, *HNFA1*, and *CTNNB1*, were detected in only one of the histologically distinct regions within the same patient (non-overlapping). Mutations in *APC* and *STK11* were each non-overlapping in two patients (Table 2). Mutations in the other seven cancer-associated genes (58%) were present in each histologically distinct region within the same patient (overlapping). Of the detected genes, the most commonly mutated gene was *TP53*, the mutations of which were detected in each histologically distinct region in four patients. Quantification of allele frequency revealed that *CTNNB1* (patient 5, epithelioid region of primary lesion) had the highest allele frequency (32.97%) among the non-overlapping genes, whereas *CDKN2A* (patient 2, primary tumor, epithelioid and spindled regions) had the highest allele frequency (83.09%) among the overlapping genes. In each case in which a *BRAF*, *NRAS*, or *KIT* mutation was identified, the mutation was present in all the histologically distinct regions. Alternatively, case 2 had no *BRAF*, *NRAS*, or *KIT* mutations.

## 3. Discussion

In this study, we analyzed mutation in 50 cancer-associated genes in melanomas exhibiting intertumor and intratumor histophenotypic variation. Our results showed that, in each patient, at least one non-overlapping mutation was detected in only one of the morphologically distinct regions, whereas one or more identical mutations were present in all of the morphologically distinct regions. Our findings indicate that histomorphologically heterogenic melanomas are often molecularly heterogenic, too. The latter may point toward a more aggressive disease and higher rates of treatment failure. Alternatively, the detection of overlapping mutations demonstrated in our current study may help pathologists accurately diagnose primary melanoma in cases with unusual histophenotypic presentations which may lead to a misdiagnosis of dedifferentiated or collision tumor, and may be useful in the diagnosis of histomorphologically heterogeneous metastases mimicking carcinoma or unclassified sarcoma [12]. Also, loss or gain of genetic alterations present only in metastatic lesions may reveal therapeutically actionable targets that can significantly impact the clinical management of patients with melanoma refractory to conventional therapy [13,14,15,16,17,18]. 

Melanoma can exhibit a variety of morphologic patterns, including rhabdoid, lipoblastic, cartilaginous, adenocarcinomatous, balloon cell, signet-ring cell, and small cell, which are often associated with several immunophenotypic alterations. However, their molecular characteristics are not well explored. The degree of the tumor molecular heterogeneity that is associated with its histologic heterogeneity is still unknown. Tumor genetic heterogeneity can occur at the level of the epigenome, genome, or transcriptome (proteome).

Although tumors originate from monoclonal cancer stem cells, as tumors evolve, tumor cells adopt new mutations and give rise to new subclones, which result in both spatial and temporal heterogeneity [19,20,21,22]. In melanoma, variable rates of mutations in cancer-related genes have been detected, ranging from 0.1 mutations per Mb to 100.0 mutations per Mb [23]. In our present study, we found that, in 80% of cases (*n* = 4) in which a *BRAF*, *NRAS*, or *KIT* mutation was identified, the mutation was present in all histologically distinct regions. These findings support the idea that the driver mutations are more conserved than passenger mutations in metastatic lesions. Most of the mutations other than *BRAF*, *NRAS*, and *KIT* mutations that we detected in metastatic lesions seemed to be associated with promotion of disease progression. These findings are similar to those of a prior study by Harbst et al. [24], who examined eight melanomas for genetic heterogeneity and found that 12% of the detected cancer-associated mutations were heterogeneously distributed, whereas *BRAF* and *NRAS* mutations, when detected, were homogeneously distributed among all the sampled regions.

In our current study, the quantification of mutant allele frequency (MAF) revealed that *CDKN2A* (patient 2, primary tumor, epithelioid and spindled regions) had the highest allele frequency (f = 83.09), suggesting loss of heterozygosity (LOH) of this gene. These findings are supportive of those of several recent studies, leading to the conclusion that MAF is a result of tumor heterogeneity in melanoma [25,26,27,28]. These studies examined the MAF of *BRAF* mutation in melanoma and revealed that the rates ranged from 0% to 97%.

Our study demonstrates that, in cases of melanoma metastasis, there may be at least one new mutation in the metastatic tumor in addition to the mutations detected in the primary melanoma. Our findings are similar to previous findings of tumor heterogeneity at the genetic level, which can alter the treatment response. Sakaizawa et al. [29] reported intertumor heterogeneity in *BRAF* mutation in circulating melanoma cells. Similarly, Lin et al. studied the MAF of *BRAF* mutation in three paired cases of primary and metastatic melanomas and found that two of the three cases had a higher MAF of *BRAF* mutation in the metastatic site than in the matched primary tumor [30]. Prior studies have also detected heterogeneity in members of signaling pathways in metastatic melanoma. Casula et al. [31] observed *CDKN2A* mutation in 11 of 16 metastatic tumors but not in paired primary melanomas whereas Goswami et al. [32] observed heterogeneity in *KRAS* and *KIT* mutations between paired cases of primary and metastatic melanomas.

In melanoma, tumor genetic heterogeneity remains one of the most common reasons for resistance to therapy [33], in which certain mutations may lead to resistance to targeted therapy. For instance, in our current study, *Rac1 P29S* mutation was identified in the metastatic melanoma of patient 3, which would carry a potential risk for resistance to Raf-therapy [34]. Similarly, variable response to immune checkpoint blockade was observed in cases with high tumor heterogeneity [35]. Additionally, high tumor heterogeneity may result in multiple tumor subpopulations that harbor heterogeneous mutations in cancer-related genes, which promote tumor survival and disease progression [36].

To detect tumor heterogeneity, although various methods can be used, NGS-based analysis is considered one of the most helpful tools for detecting mutations that explain tumor heterogeneity due to its high sensitivity that can detect mutations that occur in a small percentage of tumor cells [37]. In addition to being precise and accurate, NGS-based methods are relatively cost-effective compared to other methods, such as real-time PCR and Sanger sequencing [38]. Although our analysis was limited to the 50 genes interrogated by the NGS panel, a more extended panel or the use of whole-genome sequencing may result in lower rates of heterogeneity or false homogeneity.

## 4. Materials and Methods

Formalin-fixed, paraffin-embedded tissue samples of melanomas exhibiting intertumor or intratumor histologic heterogeneity were obtained from the archived materials of the Department of Pathology at the University of Texas MD Anderson Cancer Center over a two-year period. Sample collection and study protocols were approved by the MD Anderson Cancer Center Institutional Review Board. Intratumor histologic heterogeneity was defined as tumor with more than one distinct histologic pattern but maintaining well-formed boundaries, whereas intertumor histologic heterogeneity was defined as paired samples of primary and metastatic melanomas with completely distinct and non-overlapping mutually exclusive histologic patterns. A cutoff of 1.0 mm Breslow thinness and 1.0 mm in diameter of tumor deposit were used as criteria for sampling adequacy of primary and metastatic melanomas, respectively. To limit the sampling error using laser capture microdissection (LCM), melanomas exhibiting intermingled or overlapped histomorphologies in primary and metastatic lesions were excluded. For unequivocal histologic and phenotypic interpretation, tumors with heavy melanin pigmentation were also excluded from the study. After application of these selection and exclusion criteria, primary tumors from 2 patients and paired primary and metastatic tumors from 3 patients yielded 8 tumors and 11 histomorphologically distinct regions were identified. Whenever feasible, the immunohistochemical profile of the retrieved cases was also obtained to permit comparison of melanoma marker expression among the histologically distinct regions. Additional immunohistochemical staining for melanocytic markers, including S100 protein, SOX10, HMB-45 antigen, Melan-A, and pan-melanocytic cocktail, were performed in our Clinical Laboratory Improvement Amendments (CLIA)-certified laboratory on all the samples with sufficient tissue to complete the immunohistochemical whenever needed. Tissue blocks were sectioned at 5 μm thickness, placed on uncharged slides, and stained with hematoxylin and eosin. After analysis of the histomorphology, tumor tissue was laser microdissected for NGS analysis.

### 4.1. Laser Capture Microdissection and DNA Extraction

From each of the 11 histomorphologically distinct regions, an area in which the neoplastic melanocytes compromise at least 90% of the cells, was obtained by LCM. Approximately 4 × 10 μm^3^ of tissue per sample was captured after adjustment of the laser amplitude, pulse duration, and number of hits. DNA was isolated by treating the tissue samples with 100 μL of 0.04% proteinase K at 42 °C overnight followed by heat-denaturing at 95 °C for 10 min. The genomic DNA extracted from each microdissected area was subjected to NGS-based analysis for the detection of somatic mutations in the coding sequence of 50 genes.

### 4.2. Test Platform

Polymerase chain reaction (PCR)-based sequencing was performed in a CLIA-certified laboratory using NGS platform on genomic DNA to screen for mutations in the coding sequences of the following 50 cancer-associated genes: *ABL1, AKT1, ALK, APC, ATM, BRAF, CDH1, CDKN2A, CSF1R, CTNNB1, EGFR, ERBB2, ERBB4, EZH2, FBXW7, FGFR1, FGFR2, FGFR3, FLT3, GNA11, GNAQ, GNAS, HNF1A, HRAS, IDH1, IDH2, JAK2, JAK3, KDR, KIT, KRAS, MET, MLH1, MPL, NOTCH1, NPM1, NRAS, PDGFRA, PIK3CA, PTEN, PTPN11, RB1, RET, SMAD4, SMARCB1, SMO, SRC, STK11, TP53*, and *VHL*. NGS was performed using the Ampliseq Cancer Hotspot Panel version 2 in an Ion S5 system. Analysis was performed using Torrent suite version 5.6 with GRCh37/hg19 as the reference genome. Adequately covered amplicons were defined as those having total coverage depth of at least 250 reads.

We determined the effective lower limit of detection of this assay (analytical sensitivity) for single nucleotide variations to be in the range of 5% (1 mutant allele in the background of 19 wild-type alleles) to 10% (1 mutant allele in the background of 9 wild-type alleles) by taking into consideration the depth of coverage at a given base and the ability to confirm low-level mutations using independent conventional platforms. The NGS-based study was targeted to detect mutations in cancer-related genes and genes related to signaling pathways that can affect tumor cell viability, promote disease progression, and predict response to therapy.

## 5. Conclusions

Our present study confirmed that melanomas exhibiting morphologic heterogeneity are often molecularly heterogeneous neoplasm. In each patient, one or more identical driver mutations were present in each of the morphologically distinct regions, and at least one genomic alteration in a gene not usually associated with melanoma was present in only one of the morphologically distinct regions. These findings support performing comprehensive molecular profiling beyond the major mutations of melanoma (*BRAF, NRAS*, and *KIT*) to detect rare cancer driver genes. Currently, NGS is the most reliable and cost-effective method for evaluating tumor heterogeneity. Genetic heterogeneity may reveal therapeutically actionable targets that can significantly impact the clinical management of patients with melanoma refractory to conventional therapy.

## Figures and Tables

**Table 1:** Demographic and clinical characteristics of the patients with melanomas analyzed by next-generation sequencing (NGS). Abbreviations: SLN, sentinel lymph node; M, male; WLE, wide local excision; F, female.

Patient	Age/Sex	Tumor Site(s)	SLN Metastasis	Treatment and Disease Outcome
		***Primary lesion only***		
1	79/M	Left occipital scalp	No	WLE with negative margins. Multiple metastases in the lung detected 5 months after the initial diagnosis. Pembrolizumab 200 mg/mm^2^ + albumin-bound paclitaxel (Abraxane) 200 mg/mm^2^ for 6 one-month cycles. Hepatic and ischial bone metastases detected 11 months after the initial diagnosis. At last follow-up, 21 months after the initial diagnosis, the patient was alive with stable metastatic disease.
2	70/M	Left clavicular area	No	WLE with negative margins. At last follow-up, 20 months after the initial diagnosis, the patient was disease-free.
		***Paired primary tumor and metastatic lesion***		
3	72/M	Primary: right forehead Metastasis: right intraparotid lymph node	No	WLE with negative margins. Nivolumab therapy was initiated, but the patient was lost to follow-up and died of unknown cause 12 months after the initial diagnosis.
4	81/F	Primary: plantar surface of right foot Metastasis: right groin lymph node	Yes	WLE with negative margins. Nivolumab × 6 cycles. Metastasis to right pelvic soft tissue was detected 21 months after the initial diagnosis.
5	75/M	Primary: Left lateral arm Metastasis: Left lateral forearm (in-transit)	Yes	Treatment and follow-up not available.

**Table 2:** Histopathologic, immunohistochemical, and molecular findings of the melanomas analyzed by next-generation sequencing. Abbreviations: PanMel, Pan-melanocytic cocktail; PRI, primary; MET, metastatic, LVI, lymphovascular invasion; PNI, perineural invasion; LMM, Lentigo maligna melanoma; SSM, superficial spreading melanoma; ALM, acral lentiginous melanoma; NM, nodular melanoma.

Case	Histologic Subtype	Immunohistochemical Profile						Breslow Thickness (PRI) or Largest Dimension (MET) in mm	Mitoses Per mm^2^	Ulceration Width in mm	LVI/PNI (PRI Only)	Detected Mutations
		Morphology	S100	SOX10	PanMel	HMB-45	Melan-A					
***Primary lesion only***												
1	LMM	Nevoid	+	+	+	+	+	7.1	5	-	+/+	*NRAS*, *TP53*, *APC*, *KDR*
		Spindled	+	+	+ (focal)	-	-					*NRAS*, *TP53*, *KDR*
2	SSM	Epithelioid	+	+	+	+ (patchy)	+ (patchy)	2.3	2	-	-/+	*TP53*, *CDKN2A*, *STK11*
		Spindled	+ (patchy)	+	-	-	-					*TP53*, *CDKN2A*
***Paired primary and metastatic lesions***												
3	SSM	Primary: Epithelioid	+	+	+	+	+	1.2	3	-	-/-	*BRAF*, *TP53*, *ATM*
		Metastasis: Rhabdoid	+ (patchy)	+ (patchy)	+ (patchy)	+ (focal)	-	1.3				*BRAF*, *TP53*, *ATM*, *RAC1*
4	ALM	Primary: Epithelioid	+	+	+	+	+	3.1	6	0.1	+/-	*KIT*, *HNFA1*
		Metastasis: Foamy/balloon-like	+	+	+	NA	+	60				*KIT*, *APC*
5	NM	Primary: Epithelioid	+	+	+	+	+	12	14	18	+/+	*NRAS*, *TP53*, *CTNNB1*
		Primary: Rhabdoid	+ (patchy)	-	-	-	+ (focal)					*NRAS*, *TP53*
		Metastasis: Myxoid stroma	+ (focal)	+ (patchy)	-	-	+ (weak)	1.6				*NRAS*, *TP53*, *STK11*
